# With Reference to Reference Genes: A Systematic Review of Endogenous Controls in Gene Expression Studies

**DOI:** 10.1371/journal.pone.0141853

**Published:** 2015-11-10

**Authors:** Joanne R. Chapman, Jonas Waldenström

**Affiliations:** Centre for Ecology and Evolution in Microbial Model Systems, Linnaeus University, Kalmar, Sweden; University of Lleida, SPAIN

## Abstract

The choice of reference genes that are stably expressed amongst treatment groups is a crucial step in real-time quantitative PCR gene expression studies. Recent guidelines have specified that a minimum of two validated reference genes should be used for normalisation. However, a quantitative review of the literature showed that the average number of reference genes used across all studies was 1.2. Thus, the vast majority of studies continue to use a single gene, with β-actin (*ACTB*) and/or glyceraldehyde 3-phosphate dehydrogenase (*GAPDH*) being commonly selected in studies of vertebrate gene expression. Few studies (15%) tested a panel of potential reference genes for stability of expression before using them to normalise data. Amongst studies specifically testing reference gene stability, few found *ACTB* or *GAPDH* to be optimal, whereby these genes were significantly less likely to be chosen when larger panels of potential reference genes were screened. Fewer reference genes were tested for stability in non-model organisms, presumably owing to a dearth of available primers in less well characterised species. Furthermore, the experimental conditions under which real-time quantitative PCR analyses were conducted had a large influence on the choice of reference genes, whereby different studies of rat brain tissue showed different reference genes to be the most stable. These results highlight the importance of validating the choice of normalising reference genes before conducting gene expression studies.

## Introduction

The use of real-time quantitative PCR (RT-qPCR, hereafter abbreviated to qPCR) to quantify mRNA transcription has revolutionised our understanding of cellular responses triggered by the developmental progression or experimental manipulation of individuals [1]. As qPCR is an extremely sensitive method that allows detection of small dynamic changes in gene expression between samples, it is imperative that due care is used in every step of sample preparation and processing. When properly employed, data normalisation helps to correct for inevitable experimental variations, the most common of which are disparities in the amount of starting material [2] and/or sample loading [3].

While several methods of data normalisation are available (reviewed in [4–6]), the most commonly employed is the use of stably expressed reference genes (RGs) as endogenous (*i*.*e*. internal) controls. This method is essential when results are expressed in terms of relative quantification (fold-change) using the popular 2^-ΔΔCT^ method [7] or modified versions thereof (*e*.*g*. [8–11]). Thus, an essential step in development of qPCR assays is identification of appropriate RGs for normalisation of the data. Crucially, the RG must be unaffected by the experimental treatment(s) and/or developmental stages under investigation. Thus, identifiable changes in expression of the genes(s) of interest (GOI(s)) must not be biased by changes in expression of the RGs between experimental groups. As correct normalisation is such an integral component of gene expression analysis, it has been strongly advocated that at least two RGs be employed and the geometric mean of RG expression levels be used for normalisation (*e*.*g*. [2, 4, 11]).

The most frequently chosen RGs are so called ‘housekeeping genes’–being those required for basic cellular processes. To constitute an ideal RG, a gene must display constitutive, stable expression in all cell types/tissues and treatment regimens under consideration. It should also be expressed at similar levels (as indicated by comparable Cq values) as the GOI. The Cq, or quantification cycle, (also known at the C_T_, or threshold cycle) is the number of cycles of amplification required to reach a pre-selected threshold of fluorescence. In vertebrate studies, glyceraldehyde 3-phosphate dehydrogenase (*GAPDH*), β-actin (*ACTB*) and 18S rRNA ribosomal RNA (*18S rRNA*) have historically been popular choices [12]. However, it is becoming increasingly apparent that the utility of these genes is limited in many cases due to differential expression across species, tissue types, cell lines, developmental stages and/or in response to experimental treatments (*e*.*g*. [11–14]). If gene expression analyses are normalised against RGs that fluctuate under the experimental conditions used, then small but potentially biologically relevant changes in expression of the GOI(s) of interest can easily be missed or overemphasised [15]. For example, using unstable RGs to normalise can result in overestimating (*e*.*g*. [16, 17]) or underestimating (*e*.*g*. [18, 19]) GOI expression levels. Several methods are available for testing RG stability. Currently, the most popular [5, 20] are the BestKeeper [21], geNorm [11] and NormFinder [22] programmes. While the algorithms employed by each of these methods differs somewhat (reviewed in [23]), all are based on estimating the variance in Cq values of each RG tested across treatment groups.

In February 2009, the Minimum Information for Publication of Quantitative Real-Time PCR Experiments (MIQE) guidelines were published [24]. These guidelines lay the foundation for the optimum methodology in gene expression studies to ensure reliable and repeatable results, including a comprehensive checklist of procedures that should be followed encompassing sample preparation and handling, quality control, cDNA creation, qPCR and data analysis. With respect to RG selection, the key recommendations are that two or more RGs should be employed and the choice of RGs should be validated to ensure stable expression across treatment groups for the given experimental setting and sample set. Despite the widespread dissemination and advocacy of these guidelines, it is currently unclear to what extent these recommendations have been implemented in practical terms.

Here, we undertake two systematic quantitative reviews of the gene expression literature to assess current trends in the selection and use of RGs in: (1) studies quantifying expression of GOI(s); and (2) studies screening a panel of RGs to determine those with highest stability. We posited that over time, as the MIQE guidelines [24] have become more widely disseminated, fewer studies would be published without first providing evidence that RG stability had been quantified, increasing numbers of RGs would be tested for stability, and the use of *GAPDH* and *ACTB* as normalisers would become less widespread.

## Materials and Methods

**Fig 1:**
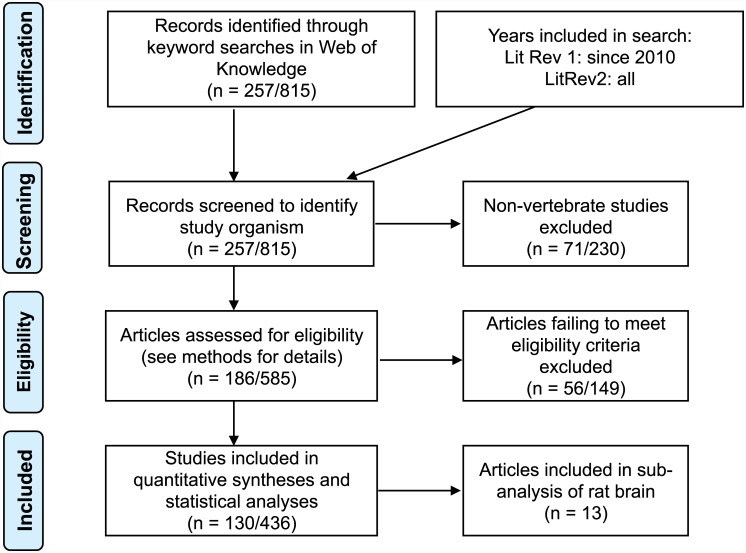
PRISMA flowchart. The number (n) of studies included and excluded at various stages of the quantitative review process, where numbers are specified as LitRev1/LitRev2.

Two quantitative literature reviews (LitRev) were conducted to assess the current methodology with respect to RG selection and use in qPCR studies. We used the Preferred Reporting Items for Systematic Reviews and Meta-Analyses (PRISMA) guidelines [25] for reporting methodology (Fig 1, S1 Table). The full tables of papers used and data extracted are provided as Supplementary Information (S2–S4 Tables).

### 1) LitRev 1: Current patterns of RG use in gene expression studies

To determine whether the MIQE guidelines are being adhered to, we conducted a restricted literature review on 10 July 2015 of gene expression studies in the years following their publication. The search was restricted to papers published since 2010, to allow one year for dissemination of the guidelines and implementation by researchers. As a vast number of gene expression papers are published every year, the search (conducted in Web of Science (WoS), Thomson Reuters) was restrictive, as follows: TITLE: "gene expression", TOPIC: “real-time quantitative PCR” and limited to TYPE: “article”. While this restricted search meant that many gene expression studies were excluded from analysis, we felt that it provided an unbiased overview of current gene expression studies.

The returned papers (*n* = 257) were manually searched and papers retained if they met the following three additional criteria: (1) the study was conducted on a vertebrate species; (2) we had access to the full-text article; and (3) the paper reported the results of a gene expression analysis and was not solely a RG selection paper (such papers were analysed in LitRev 2, described below). Of the papers retained (*n* = 128), the methodology and results were searched to determine: (1) the number of RGs used; (2) the identity of the RG(s); (3) the number of GOIs subsequently analysed; and (4) whether there was evidence that the authors had tested a panel of RGs to select those with the most stable expression, either in the current work or in previous work conducted in the same lab with the same/similar samples.

### 2) LitRev 2: Testing panels of RGs for expression stability

Our second literature review focused on papers specifically testing panels of RGs to determine their stability. Papers that subsequently included analysis of GOI expression were included, but this was not a pre-requisite for inclusion in LitRev2 (in contrast to LitRev1). The aim was to determine whether methodology is changing over time or differs between fields (such as model versus non-model species) and the consequences this has on the number and identity of RGs tested and selected. We conducted a literature search on 15 July 2015 in WoS using the following search string: TOPIC: "Gene expression" and TOPIC: “bestkeeper OR genorm OR normfinder” and limited to TYPE: “article”. All years were included.

The returned results (*n* = 815) were manually searched and studies retained if they fit the following criteria: (1) they were conducted on a vertebrate species; (2) they did not solely test micro-RNA (miRNA) RGs; and (3) they used qPCR to test a panel of at least three RGs (thus, papers using one of the software programs solely on micro-array data were eliminated). Many of the retained papers (*n* = 436) included analyses on multiple tissues or treatments. These studies were listed once in our table of results to avoid pseudo-replication. However, some papers (*n* = 17) provided results for multiple species. The results for each different species were recorded separately as there is no *a priori* reason to expect the same RGs to be optimal in different species. Thus, our final dataset comprised 457 results from 436 studies. Of the papers retained, the methodology was searched to determine: (1) the number of RGs used; (2) the study species; (3) whether *GAPDH*, *ACTB* or *18S rRNA* were included in the panel tested; and (4) if so, whether these genes were subsequently selected within the most stable (two to four) genes required for accurate normalisation. For point (1), three studies used different numbers of RGs for different tissues or treatments. In such cases, we took the largest number of RGs tested in a single analysis as our number of RGs tested. For point (4), we used the authors’ recommendations of which RGs provided the best normalisation for the given tissue and/or experimental treatment or the number of genes required to obtain a stability V value below 0.15 in GeNorm as suggested by Vandesompele *et al*. [11]. In some cases, authors simply listed stability values for each gene without providing a recommendation. In such cases, we searched the results to determine which two to four genes had the highest stability for the given tissue and/or experimental treatment. By default, we selected the two most stable unless the authors showed that three or four RGs were required to reach a threshold stability value. In cases where the authors showed that five or more RGs would be required to provide reliable normalisation, we considered that no RGs were optimal—given the methodological and financial limitations of normalising with such a high number of RGs.

In order to provide a more comprehensive examination of the RGs tested and selected within a single tissue type from one species, we determined that the most common non-human tissue examined in LitRev 2 was rat brain. We chose to exclude human cell-line studies from this analysis because tissue samples were the more common source of RNA across all studies, and likely represent a more homogeneous source material than cell-line extracts. We examined these studies further to determine if different studies, with different treatment regimens and/or different laboratory methods, resulted in the same RGs being chosen. This should be the case if RGs are universally stable for a given tissue, independent of treatment effects and differences between laboratories. We classified studies by the structure(s) of the brain examined and treatment regime(s) and determined the RGs tested and selected. Some studies analysed their data with more than one stability testing algorithm. In such cases, we preferentially recorded the results from the GeNorm analysis in the main analysis (11/13 studies) and noted when other algorithms provided differing results as Supplementary Information (S4 Table). When provided, we listed all genes required for stability based on V values falling below the standard threshold of 0.15 [11]. When this analysis was not provided we listed the two most stable genes. We excluded studies that performed analyses on rat cell lines (*n* = 3) and those that included both mRNA and microRNA RGs in the same analysis (*n* = 1). This resulted in 25 results from 13 studies being retained in the analysis.

### Statistical analyses

#### LitRev 1: Current patterns of RG use in gene expression studies

We assessed the influence of time on the number of RGs utilised via a generalised linear model (GLM) with Poisson errors since the response variable was count data, and deviance goodness of fit to assess the fit of the model. We used a Mann-Whitney U test to determine whether studies that test a panel of RGs for stability subsequently use more RGs when testing expression of the GOI than those that do not. The use of a non-parametric test was required as the response variable (number of RGs utilised) did not fit normality in this dataset. We investigated the influence of the number of GOIs investigated on the amount of effort expended in testing and using reference genes via GLMs with the following three response variables and associated error distributions: 1) whether or not RG stability was tested (binomial error); 2) the number of RGs used to normalise data (Poisson error); and 3) the number of RGs included in the panel amongst the subset of studies that tested RG stability (Poisson error).

#### LitRev 2: Testing panels of RGs for expression stability

**Fig 2:**
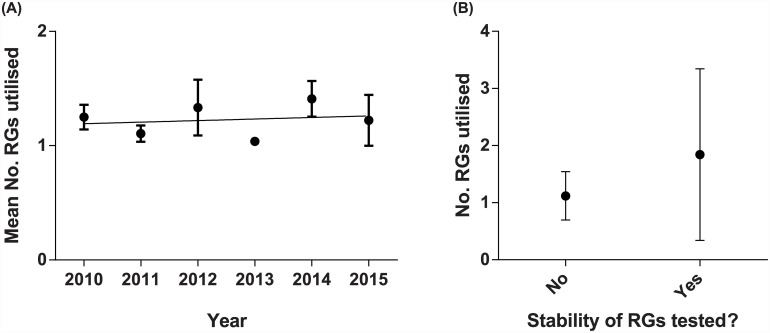
Publication of studies testing expression of GOI(s). Papers included in LitRev1 showing (A) The mean number (no.) of RGs utilised in gene expression studies per year. (B) Mean no. of RGs utilised, according to whether or not a panel of RGs was first tested for stability. In both cases, data is represented as mean (dot) ± standard deviation (bars).

**Fig 3:**
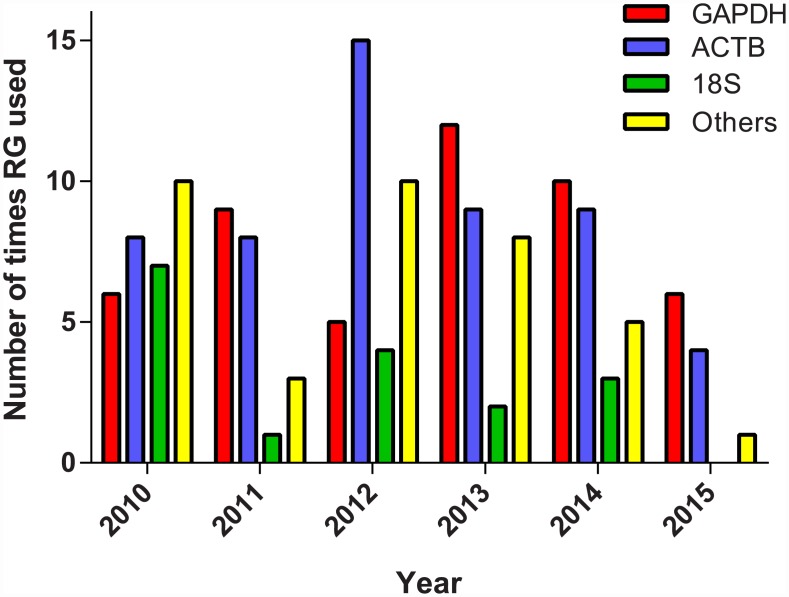
Utilisation of *GAPDH* and *ACTB* and *18S rRNA* as RGs in gene expression studies since the publication of the MIQE guidelines.

**Fig 4:**
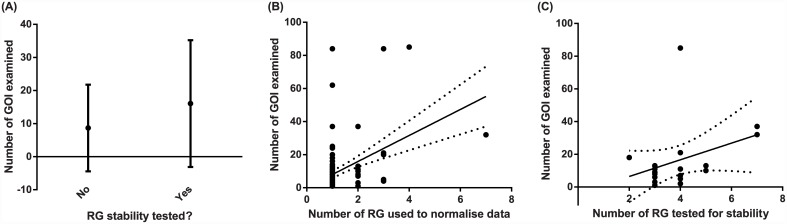
Influence of the number of GOI investigated on the rigor with which RGs are selected and used. (A) Number of GOIs analysed does not affect whether or not the stability of RGs is tested. (B) Number of GOIs analysed does not affect the number of RGs used to normalise data. (C) Amongst those studies that do test a panel of RGs for stability (*n* = 19), more RGs are tested in studies subsequently analysing more GOI. For panels (B) and (C), a linear regression (solid line) and 95% confidence intervals (dotted lines) were fitted solely to show the direction of the relationship.

**Fig 5:**
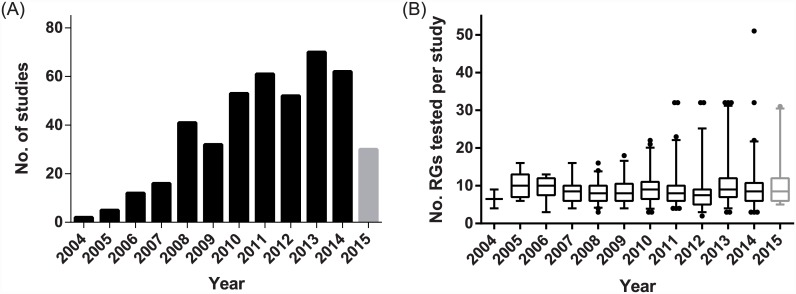
Publication of studies testing a panel of RGs for expression stability. Papers included in LitRev2 showing (A) Temporal trend, and (B) Median and interquartile range of number (no.) of RGs included in stability panels per year, where whiskers span the 5–95 percentiles. Data for 2015 are shown in grey as this comprises the months January to June 2015 only.

**Fig 6:**
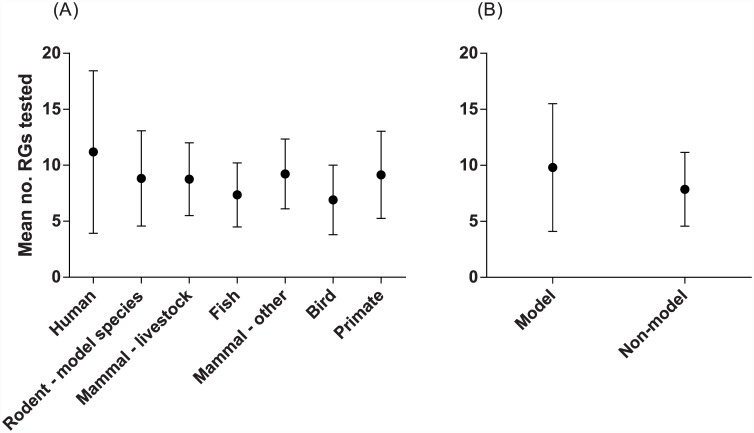
Number of RGs tested for stability based on taxonomic categorisation of study organism. (A) Number of RGs used per species grouping. (B) Number of RGs used in model and non-model species. In both cases, data is represented as mean (dot) ± standard deviation (bars).

We investigated the influence of time on the number of RGs included in panels for stability testing using a linear model after log transforming the response variable (number of RGs tested). This was owing to the fact that we could not use Poisson regression due to overdispersion of the data. In this case, we used a Kolmogorov-Smirnov test [26] to assess the Gaussian error distribution. We used GLMs with binomial errors to determine whether the selection of *GAPDH*, *ACTB* or *18S rRNA* as a stable RG was influenced by the number of RGs included in the panel and deviance goodness of fit to assess the fit of the model. For these tests, we first recorded whether *GAPDH*, *ACTB* or *18S rRNA* was included amongst the panel of RGs in each paper as a categorical variable with four possible outcomes: *NA* (not tested), *yes* (tested and selected), *no* (tested and not selected) or *sometimes* (tested, selected in a subset of the treatments and/or tissue types analysed). We then only considered the variables *yes* and *no* (binomial response variable) in the models. We assessed whether the number of RGs tested differs with respect to the type of species the study was conducted on in two ways. Firstly, we used a Kruskal-Wallis test followed by Dunn’s post-hoc test for multiple unequal group comparisons to determine whether broad species groupings differed. To facilitate this, we first produced a seven-level categorical variable for the type of species the study was conducted on, as follows: *human*; *non-human primate*; *rodent model* (rat, mouse, guinea pig); *livestock mammal* (cow, pig, sheep, goat, horse, buffalo, yak, deer); *other mammal* (dog, cat, rabbit, wild rodent, dolphin); *bird*; *fish*. Secondly, we used a Mann-Whitney U test to determine whether the number of RGs used differs between model and non-model organisms. The use of non-parametric tests (Kruskal-Wallis and Mann-Whitney) were required because the response variable (number of reference genes) is count data and thus does not follow a Gaussian distribution. This classification was based on whether there is a full genome sequence available for the study species in the Ensembl database (http://www.ensembl.org/info/about/species.html) on 30 July 2015. Species with a full genome sequence available were classified as *model* species and the remainder were classified as *non-model* species. While undoubtedly some of the studies on model species were published before the availability of their genome sequence, we felt that this classification provided a proxy for the amount of molecular research conducted on the species and therefore, the availability of genetic resources such as primers. With the exception of non-parametric tests, all statistical analyses were performed in R v 3.0.2 [27]. Non-parametric tests and plots were conducted in GraphPad Prism v.6.03 (GraphPad Software Inc, San Diego, CA, Figs 2–6) or R (S1 Fig).

## Results

### LitRev 1: Current patterns of RG use in gene expression studies

The average number of RGs used was 1.23 (*n* = 128; range 1–7) with only 13% (17/128) of studies using more than a single RG for normalisation (Fig 2A). Furthermore, there was no evidence that the number of RGs used for normalisation increased over time (test for the slope: *z* = 0.206, *P* = 0.837). Only 19 studies (15%) provided evidence that a panel of RGs had been tested to find those with stable expression. Amongst these, the mean number of RGs tested was 3.9 (range 2–7). Studies that tested a panel of RGs for stability subsequently used more RGs in their analyses of gene expression than those that did not (two-tailed Mann-Whitney *U* = 683.5, *P* = 0.0003, *n* = 128, Fig 2B). However, such studies continued to use very few RGs for normalisation of qPCR data (mean = 1.84, range 1–7 versus studies that did not test a panel: mean = 1.10, range = 1–3). Amongst all included studies, the most common choice of RGs were *ACTB* (used in 38% of the studies) and *GAPDH* (37%) with *18S rRNA* (12%) being somewhat less utilised (Fig 3).

We further tested whether the number of GOIs investigated influenced the research effort expended finding suitable RGs. We found that the number of GOIs analysed was marginally unrelated to whether or not stability of RGs was tested (test for the slope: *z* = 1.887, *P* = 0.059, *n* = 128, Fig 4A). However, there was a significant relationship between the number of RGs used to normalise data and the number of GOIs subsequently tested (test for the slope: *z* = 2.960, *P* = 0.003, *n* = 128, Fig 4B), and amongst the 19 studies that did test for RG stability, the number tested in the panel was influenced by the number of GOIs under investigation (test for the slope: *z* = 2.740, *P* = 0.006, *n* = 19, Fig 4C). In both cases, the number of RGs used/assessed increased in studies subsequently analysing more GOIs.

### LitRev2: Testing panels of RGs for expression stability

The average number of RGs tested for stability was 9.53 (*n* = 456; range 2–51). An increasing number of studies testing panels of RGs for expression stability are being published (Fig 5A), but we found no evidence that amongst these studies an increasing number of RGs are included in the panel over time (test for the slope: *t*_456_ = 1.412, *P* = 0.159, Fig 5B). *GAPDH* and *ACTB* were commonly included in the panel of genes tested (*GAPDH* = 89%, *ACTB* = 86%); in contrast, *18S rRNA* was included in slightly less than half (48%) of the studies. Importantly, we found that there was a significant relationship between the number of RGs tested and the likelihood that *GAPDH* (*z* = -5.113, *P* < 0.001, *n* = 307), *ACTB* (*z* = -3.506, *P* < 0.001, *n* = 328) or *18S rRNA* (*z* = -4.311, *P* < 0.001, *n* = 177) were chosen as the most stable RGs for normalisation, whereby these three genes are significantly less likely to be chosen when more RGs were screened in the panel (S1 Fig).

Most studies were conducted on *humans* (38% of studies), followed by *rodent model* species (22%) and *livestock* (19%) (Table 1A). On average, *human* studies tested the highest number of potential RGs (11.2) and *bird* studies the fewest (6.9) (Table 1A). Across all groupings, significantly different numbers of RGs were tested (Kruskal-Wallis statistic = 25.08, *P* = 0.0003, Fig 6A). Posthoc testing via Dunn’s multiple comparisons test revealed that the only significant difference between groupings was between *humans* and *fish*; whereby more RGs were tested in human studies (S5 Table). For other comparisons, some tests were underpowered due to few studies in some of the categories (Table 1A). Similarly, we found that significantly more RGs were tested for stability in studies of *model* than *non-model* species (Mann-Whitney U = 9989, *P* = 0.003, Table 1B, Fig 6B).

**Table 1:** Number (no.) of studies and reference genes (RGs) analysed according to: (A) species groupings (rodent = rodent model species, livestock = livestock mammal) and (B) model versus non-model species.

	(A)							(B)	
	*Human*	*Rodent*	*Livestock*	*Fish*	*Mammal—other*	*Bird*	*Primate*	*Model*	*Non-model*
**No. studies**	174	101	91	51	22	10	7	390	66
**Mean no. RGs tested**	11.18	8.83	8.76	7.35	9.23	6.90	9.14	9.81	7.86
**Std. Dev of mean**	7.25	4.25	3.26	2.86	3.12	3.11	3.89	5.70	3.30

**Fig 7:**
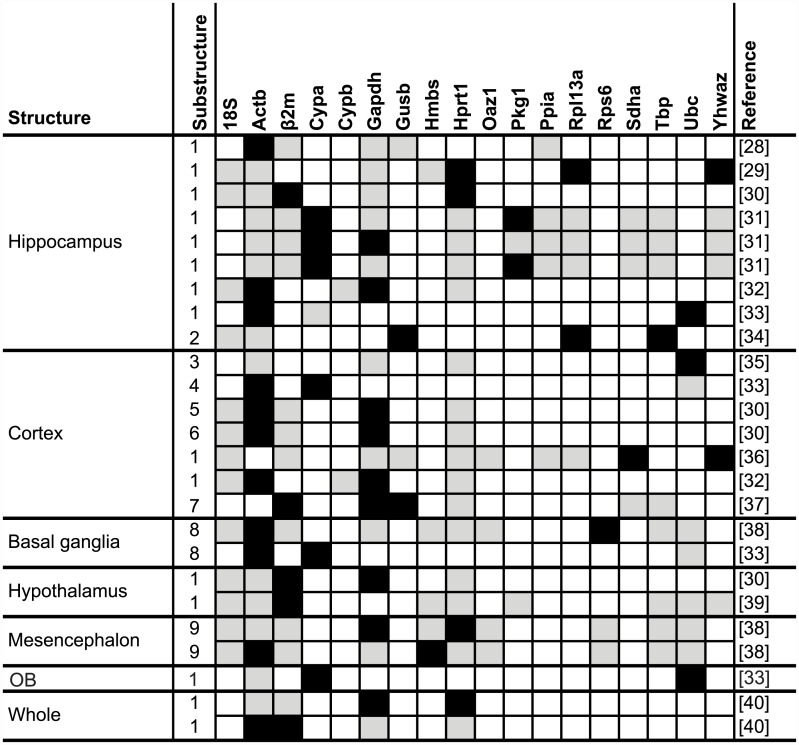
Testing and selection of RGs in different structures of the rat brain after various treatments. Shading in grey indicates the gene was tested and black indicates it was selected as within the group of most stable genes required for accurate normalisation. OB is the olfactory bulb with cerebral substructure codes provided as a footnote. Only genes tested more than once included. See S4 Table for further details of each study, including treatment regime and full list of all RGs tested and selected. ^*a*^ *Substructures*: *1 = Whole*, *2 = Dendate gyrus*, *3 = Auditory*, *4 = Prefrontal*, *5 = Frontal*, *6 = Temporal*, *7 = Whole + Basal ganglia*, *8 = Striatum*, *9 = Nigra*.

Given that a suitable RG should display stable expression independent of experimental treatment, one should expect that the same RGs will be chosen within a given species and tissue, independent of study-level effects. We tested this for a subset of LitRev2 studies focussing on rat brain (the most common non-human tissue investigated) [28–40] and found that different researchers selected different RGs even within the same cerebral sub-structures (Fig 7). Five genes were tested in more than half of the studies (*18S rRNA*, *Actb*, *β2m*, *Gapdh and Hprt1*). However, none of these popular RGs were selected as stable more than 50% of time. In fact, amongst RGs tested more than once, the most commonly selected was *Cypa* (although it should be noted that while this gene was tested in seven separate analyses, this comprised just two individual studies; Fig 7).

## Discussion

Accurate normalisation of transcripts is a fundamental requirement for gene expression studies. As such, we aimed to investigate the current state of research practice with respect to data normalisation in qPCR studies of gene expression. Taken together, these two quantitative reviews reveal there is a fundamental disconnect between studies testing panels of RGs for stability and the practical implementation of RGs in gene expression studies. While there is increasing awareness of the need to test RG stability before choosing which genes to normalise qPCR data with (LitRev 2; Fig 5A), few studies are currently converting this awareness into a more robust methodological approach when analysing the expression of GOIs (LitRev 1; Fig 2). Given that a clear set of guidelines are now available to assist researchers in performing gene expression analyses [24], the failure of the vast majority of LitRev1 studies to thoroughly investigate RG stability and use adequate numbers of RGs for data normalisation calls is surprising and concerning. Indeed, only two studies [41, 42] in LitRev1 noted that they followed the MIQE guidelines during experimental procedures. Furthermore, though papers testing panels of potential RGs for stability are increasing in number (LitRev2), the number of individual RGs tested has not significantly increased. However, there does appear to be a trend for increasing numbers of RGs to be tested in the last few years (Fig 5B). Interestingly, more effort appears to be expended on RG selection and use in those studies subsequently analysing more GOIs (Fig 4B and 4C). While this is encouraging, analysis of one or a few GOI(s) does not negate the requirement that data is first normalised with RGs that have been shown to be stable within the experimental context.

Of the 23 studies in LitRev2 that included twenty or more RGs, all have been conducted since 2010. All but one of these were conducted on model species: 18 (78%) were conducted on humans, three (13%) on model rodent species (rats and mice), and one (4%) on zebrafish. The remaining study was conducted on a less well characterised, but nevertheless domestic, species: sika deer (*Cervus nippon*). This bias towards larger numbers of RGs being tested when the study species is a model organism can likely be attributed to many more PCR primers being available in such species. Furthermore, two of the most promising avenues for robust identification of stable RGs are more tractable in model species. The first is the use of microarray data, whereby a comparative analysis of microarray results from the species (and tissue) of interest, across treatment and experimental regimes and originating from different laboratories, can identify potentially stable genes for further testing via qPCR. This approach has been increasingly employed in the last few years (*e*.*g*. [43–45]), and often results in the discovery of novel RGs for data normalisation. The RefGenes tool in Genevestigator (http://www.genevestigator.com) has been developed to help identify stably expressed RGs from publically available microarray data [20]. The second is the use of commercially available plates preloaded with primers for a range of potential RGs, such as the TaqMan^®^ Express Endogenous Control Plates and SYBR^®^ OpenArray^®^ Real-Time PCR Plates (Applied Biosystems) or the PrimePCR Assay system (BioRad). For certain model species, particularly human, rat and mouse, the use of these plates allows efficient and rapid screening of a large number of common potential RGs for expression stability within the context of a particular set of samples and experimental conditions. This approach is increasingly popular (*e*.*g*. [46–48]) and is likely to spread as such plates become available for a variety of species.

### The idea of a universal reference gene is a fallacy

Our first literature review revealed that *GAPDH* and *ACTB*, and to a lesser extent *18S rRNA*, continue to be common RG choices (Fig 3). In over 72% of the studies included (93/128), *GAPDH*, *ACTB* or *18S rRNA* were used as the single normalising gene. We show in the second literature review that, as increasing numbers of potential RGs are screened within a study, there is a decreasing chance these three genes are selected as having the most stable expression. If any of these genes were ‘universal’ endogenous controls, as they are clearly still viewed by many researchers, then they would be preferentially selected regardless of the number of other potential RGs screened. *GAPDH* is known to be stimulated by diverse biological factors ([12] and references therein), as is *ACTB* ([49] and references therein). Moreover, both *GAPDH* and *ACTB* have numerous pseudogenes in many species [50–52], adding a further potential source of error to qPCR studies, if primers have not been thoroughly tested to ensure they do not co-amplify pseudogenes in those species where they occur. The use of *18S rRNA* as a RG has also been questioned. First, ribosomal RNA (rRNA) comprises approximately 80% of total RNA [53]. Thus, rRNA transcripts (e.g. *18S rRNA* and 28S) tend to be much more abundant than mRNA transcripts (*e*.*g*. other RGs and GOIs) in cDNA generated by random oligonucleotides, and consequently have lower Cq values [54]. As recommended by Vandesompele *et al*. [11], transcript levels (and therefore Cq values) between GOIs and RGs should be similar as this ensures all transcripts are subject to the same kinetic interactions during qPCR. Second, rRNA is transcribed by RNA polymerase I while mRNA is transcribed by RNA polymerase II. Thus, mRNA and rRNA synthesis are regulated via different pathways, and changes in one may not affect the other [55]. Third, rRNA RGs can only be used when the cDNA template is reverse transcribed from total RNA using random hexamers. This is because rRNA lacks a polyA tail, and thus synthesis of cDNA with oligo d(T) primers will only amplify mRNA transcripts. It has been suggested that random hexamer primed cDNA can greatly over-estimate mRNA copy numbers [56] and that oligo dT-primed cDNA will provide a more accurate estimation of the mRNA pool [57]. Fourth, a common approach to circumvent potential genomic DNA contamination of RNA extracts is to design primers spanning introns, thus nucleic acid containing exonic regions (*i*.*e*. DNA) will fail to amplify. *18S rRNA* is devoid of introns, and as such this approach cannot be used with this marker. Fifth, as with *GAPDH* and *ACTB*, *18S rRNA* has been shown to be regulated in response to various biological stimuli (*e*.*g*. [30, 34, 46, 58]). We found no evidence for the presence of a universal RG amongst the studies included in LitRev2. The fact that *GAPDH* and *ACTB* remain popular choices is because they were popular choices as standards during the rise of Northern blotting in the 1980s and early 1990s [59]. Thus, many research laboratories were familiar with the use of these genes and primer sequences were widely available as qPCR became popular. While the methodology moved on, it appears many researchers have been slow to update their protocols and move beyond using *GAPDH* and/or *ACTB* to a thorough investigation of the expression stability of multiple potential RGs.

### Methodological and analytical pitfalls

We noted that many studies in LitRev 2 added a layer of unnecessary experimental variability by performing a single RG stability analysis across multiple tissue types (*e*.*g*. [60–64]) or cell lines (*e*.*g*. [65–67]); we even found one paper which combined multiple tissues from two different species in a single analysis [68]. This is an unsound approach, as there is no *a priori* reason to expect that the same RG will be stable across different tissues, or even cell lines from the same tissue type. Studies that perform independent analyses on different tissues, for the same species and treatment regime, nearly universally identify different RGs in different tissues/cell types as the most stable (*e*.*g*. [16, 69–71]). Indeed, it has been shown that tissue/cell type can have a larger influence on RG stability than treatment regime [72]. Furthermore, within a single tissue, developmental stage can influence RG selection (*e*.*g*. [73–75]). Studies that perform analyses on both pooled tissues and single tissue extracts tend to reveal much higher GeNorm V values in the tissue mixture analyses—in other words they find that more RGs are required for accurate normalisation when disparate tissues are pooled (*e*.*g*. [76–78]). As such, studies comprising multiple tissue types or cell lines should either test RG stability in every tissue independently, or use RG independent normalisation methods (*e*.*g*. [5, 79, 80]). In our analysis of studies testing RGs in rat brains, we found little consensus over the choice of the most stable RGs, even within cerebral substructures (Fig 7). While *Actb*, *Gapdh* and *18S rRNA* were commonly tested in these studies, they were each selected less than half the time, showing that they cannot be considered universal RGs. Similarly, studies on different cell lines from the same tissue source tend to find different optimal RGs (*e*.*g*. liver cell lines [81]), as do studies on the same cell line performed by different researchers (*e*.*g*. HepG2 cell line [81, 82]). Unless a primary study of gene expression specifically aims to assess the differences in gene expression across multiple tissues/cell lines or treatments in a single analysis, we would strongly advocate that RG selection is performed separately for each different tissue/cell line and treatment regime under investigation. Indeed, RG selection should be performed amongst the exact same pool of samples that will be compared in the analysis of GOI expression. Thus, one can be confident that the correct normalisation has been applied for their given set of experimental conditions and samples.

### Perspectives

The incorrect use of normalising genes can have a profound effect on the conclusions drawn from studies of gene expression [15, 57, 83]. Studies that test the use of stable versus unstable RGs in the estimation of GOI expression show that the use of unstable RGs nearly universally results in spurious estimation of GOI expression (*e*.*g*. [18, 19, 84]). Yet our first literature review clearly demonstrates that many researchers continue to use a single, unvalidated reference gene to normalise data (Fig 2). One of the justifications often appears to be that the search for, and use of, multiple reference genes is cost prohibitive. We argue that this cost is insignificant compared to the cost of publishing erroneous results, given that this can lead to future research being predicated on incorrect or biased data. Given that many such studies are performed in the field of medical research, the consequences could also have negative impacts on drug development and human health. Furthermore, it is clear that validation of a RG as stable given the particular conditions of one laboratory may not mean that this RG can automatically be considered to be stable for the given species and tissue/cell type when used in other laboratories (*e*.*g*. Fig 7). We advocate that researchers should routinely be testing the stability of a large panel of potential RGs (>10, preferably >20) for use as endogenous controls before commencing any study of gene expression. Additionally, newly available methods and analytical software for normalising qPCR data (*e*.*g*. [5, 79, 85–87]) should be investigated where appropriate. Our second literature review revealed that studies specifically testing RG stability are increasing over time (Fig 5). Given our previous statement (that RGs should be tested within the context of one’s own samples); one might question the utility of publishing such studies. However, we would advocate that reference gene stability studies continue to be published, as they provide a useful starting point to identify which RGs might be appropriate to include in a test panel for their species and experimental set-up as well as primers and methodological details that can be invaluable to others. This is especially the case for non-model species, which are currently lagging behind well characterised species with respect to the number of RGs tested for stability (Table 1b; Fig 6b). Furthermore, the publication of such studies allows identification of patterns when research is synthesised quantitatively, such as the fact that selection of *GAPDH*, *ACTB* and *18S rRNA* is highly dependent on the number of potential RGs screened. Given the numerous potential pitfalls that exist when undertaking qPCR studies [59], it can seem an overwhelming task to obtain reliable results. We would argue that so long as care and attention is taken when choosing which RGs with which to normalise data, one of the major hurdles can be overcome.

## Supporting Information

S1 Fig Likelihood that three common RGs are selected as amongst the most stability expressed. (A) *GAPDH*, (B) *ACTB* and (C) *18S rRNA*. All three genes are less likely to be selected as stable when more RGs are tested.
(EPS)

S1 Table PRISMA summary. Details and locations within the text of specific components of the quantitative reviews conducted in this paper, based on the 2009 PRISMA group guidelines.
(DOC)

S2 Table LitRev1 studies. Full details of data extracted and citations for all studies included in LitRev1.
(XLSX)

S3 Table LitRev2 studies. Full details of data extracted and citations for all studies included in LitRev2.
(XLSX)

S4 Table Studies of gene expression in rat brains. Full details of the rat brain studies summarised in Fig 7, including the strain and sex of rats used, the substructure of the brain analysed, the experimental treatment applied/investigated, the reference genes (RGs) tested and selected, the number (no.) required to reach a threshold stability value of below 0.15 in GeNorm (NA = this analysis not presented) and selected notes. Results summarised preferentially based on GeNorm results.
(DOCX)

S5 Table Dunn's test for posthoc multiple unequal group comparisons. Differences in the number of RGs used in each of the seven categories of study species (rodent = rodent model species and livestock = livestock mammal).
(DOCX)
