# Supplementary material for: With Reference to Reference Genes: A Systematic Review of Endogenous Controls in Gene Expression Studies
Source: PLoS One. 2015 Nov 10;10(11):e0141853. doi: 10.1371/journal.pone.0141853 (PMC4640531; doi:10.1371/journal.pone.0141853)
Supplement: S4 Table — Full details of the rat brain studies summarised in Fig 7, including the strain and sex of rats used, the substructure of the brain analysed, the experimental treatment applied/investigated, the reference genes (RGs) tested and selected, the number (no.) required to reach a threshold stability value of below 0.15 in GeNorm (NA = this analysis not presented) and selected notes. Results summarised preferentially based on GeNorm results. (DOCX) [file pone.0141853.s005.docx]

**Table S3.**

| **Reference** | **Rat strain** | **Sex** | **Brain tissue** | **Treatment** | **RGs tested** | **RGs selected** | **No. needed for stabilisation** | **Notes** |
| --- | --- | --- | --- | --- | --- | --- | --- | --- |
| [[1](#_ENREF_1)] | Flinders | Male | Hippocampus | Methylene blue | 18s, Actb, CycA, Gapdh, Hmbs, Hprt1, Rpl13A, Ywhaz | CycA, Ywhaz, Rpl13, Hprt1 | 4 | Only 2 needed via Normfinder |
| [[2](#_ENREF_2)] | Fischer 344 | Male | Auditory cortex | Age (young vs old) | Actb, EF1α, Gapdh, Hprt1, Ubc | EF1α, Ubc | NA |  |
| [[3](#_ENREF_3)] | Sprague-Dawley | Male | Cortex and basal ganglia | Collagenase | β2m, Gapdh, Gusb, Hprt, Pol2r, Sdha, Tbp | β2m, Gusb, Gapdh, Pol2r | 4 | Across all samples. Subanalyses also performed |
| [[4](#_ENREF_4)] | Fischer 344 | Male | Cortex | Cerebral ischemia | 18s, β2m, Gapdh, Gusb, Hprt, Oaz1, Ppia, Rpl13a, Sdha, Ywhaz | Sdha, Ywhaz | 2 | Only 3 tested via GeNorm |
| [[5](#_ENREF_5)] | Sprague-Dawley | Male | Striatum | Methamphetamine + 2 or 24 hr | 18s, Actb, β2m, Gapdh, Hmbs, Hprt1, Oaz1, Rps6, Tbp, Ubc | Actb, Rps6 | 2 |  |
|  |  |  | Nigra | Methamphetamine + 2hr |  | Gapdh, Hprt | 2 |  |
|  |  |  | Nigra | Methamphetamine + 24hr |  | Actb, Hmbs | 2 | Different results with Normfinder |
| [[6](#_ENREF_6)] | Wistar-Hannover | Male | Hippocampus  Hypothalamus | Intermittent hypoxia | Gapdh, Hprt118s, Actb, β2m, | Hprt, β2m  β2m, Gapdh | NA  NA |  |
|  | |  | Frontal cortex  Temporal cortex |  |  | Actb, Gapdh  Actb, Gapdh | NA  NA | Different results with BestKeeper |

| [[7](#_ENREF_7)] | Several | Male | Hippocampus | 4 dp cardiac arrest ± minocycline | Actb, β2m, CypA, Gapdh, Hprt1, Pgk1, Ppia, Rpl13a, Sdha, Tbp, Ywhaz | CypA, Pgk1 | 2 |  |
| --- | --- | --- | --- | --- | --- | --- | --- | --- |
|  |  |  |  | 7 dp cardiac arrest ± minocycline |  | CypA, Gapdh | 2 |  |
|  |  |  |  | 21 dp cardiac arrest ± minocycline |  | CypA, Pgk1 | 2 |  |
| [[8](#_ENREF_8)] | Wistar-Hannover | Male | Whole brain | Sleep deprivation 96 hr | Actb, β2m, Gapdh, Hprt1 | Gapdh, Hprt | NA |  |
|  |  |  |  | Sleep deprivation 96 hr, + 24 hr |  | Actb, β2m | NA |  |
| [[9](#_ENREF_9)] | Long-Evans | Male | Hypothalamus | Dietry fat intake | 18s, Actb, β2m, Hmbs, Hprt1, Pgk1, PpiB, Rpl0, Rpl2, Rpl32, Tbp, Ubc, Ywhaz | β2m, Rpl0 | 2 | Across all samples. Subanalyses also performed |
| [[10](#_ENREF_10)] | Wistar | Male | Hippocampus | Pilocarpine | Actb, β2m, Gapdh, Gusb, Polr1a, Ppia, Rplp1, Tubb2a | Actb, Rplp1 | 2 |  |
| [[11](#_ENREF_11)] | Wistar | Female | Prefrontal cortex | No treatment | Actb, CypA, UbC | Actb, CypA | NA | Only NormFinder used |
|  |  |  | Hippocampus |  |  | Actb, CypA | NA |  |
|  |  |  | Olfactory bulb |  |  | UbC, CypA | NA |  |
|  |  |  | Striatum |  |  | Actb, CypA | NA |  |
| [[12](#_ENREF_12)] | Sprague-Dawley | Male | Hippocampal dentate gyrus | Febrile seizures induced by heat | 18s, Actb, Arbp, CycA, GusB, Rpl13A, Tbp | CycA, Rpl13A, Tbp, Arbp, GusB | 5 | Slight differences with NormFinder |
| [[13](#_ENREF_13)] | Wistar | Male | Cortex | ± dexamethasone, ± dietry restriction | 18s, Actb, CypB, Gapdh, Hprt | Actb, Gapdh | 2 | Across all samples. Subanalyses also performed |
|  |  |  | Hippocampus |  |  | Actb, Gapdh | 2 |  |

**Table S3 References**

1. Bonefeld BE, Elfving B, Wegener G. Reference genes for normalization: a study of rat brain tissue. Synapse. 2008;62(4):302-9. doi: 10.1002/syn.20496.

2. Chen J, Ruan R. Identifying stable reference genes for evaluation of antioxidative enzyme gene expression in auditory cortex and cochlea of young and old Fischer 344 rats. Acta Otolaryngol. 2009;129(6):644-50. doi: 10.1080/00016480802311015.

3. Cook NL, Kleinig TJ, van den Heuvel C, Vink R. Reference genes for normalising gene expression data in collagenase-induced rat intracerebral haemorrhage. BMC Mol Biol. 2010;11. doi: 10.1186/1471-2199-11-7.

4. Gubern C, Hurtado O, Rodriguez R, Morales JR, Romera VG, Moro MA, et al. Validation of housekeeping genes for quantitative real-time PCR in in-vivo and in-vitro models of cerebral ischaemia. BMC Mol Biol. 2009;10. doi: 10.1186/1471-2199-10-57.

5. He Y, Yu S, Bae E, Shen H, Wang Y. Methamphetamine alters reference gene expression in nigra and striatum of adult rat brain. Neurotoxicology. 2013;39:138-45. doi: 10.1016/j.neuro.2013.08.009.

6. Julian GS, de Oliveira RW, Perry JC, Tufik S, Chagas JR. Validation of housekeeping genes in the brains of rats submitted to chronic intermittent hypoxia, a sleep apnea model. PLoS One. 2014;9(10):e109902. doi: 10.1371/journal.pone.0109902.

7. Langnaese K, John R, Schweizer H, Ebmeyer U, Keilhoff G. Selection of reference genes for quantitative real-time PCR in a rat asphyxial cardiac arrest model. BMC Mol Biol. 2008;9. doi: 10.1186/1471-2199-9-53.

8. Lee KS, Alvarenga TA, Guindalini C, Andersen ML, Castro RMRPS, Tufik S. Validation of commonly used reference genes for sleep-related gene expression studies. BMC Mol Biol. 2009;10. doi: 10.1186/1471-2199-10-45.

9. Li B, Matter EK, Hoppert HT, Grayson BE, Seeley RJ, Sandoval DA. Identification of optimal reference genes for RT-qPCR in the rat hypothalamus and intestine for the study of obesity. Int J Obes. 2014;38(2):192-7. doi: 10.1038/ijo.2013.86.

10. Marques TEBS, de Mendonca LR, Pereira MG, de Andrade TG, Garcia-Cairasco N, Paco-Larson ML, et al. Validation of suitable reference genes for expression studies in different pilocarpine-induced models of mesial temporal lobe epilepsy. PLoS One. 2013;8(8):e71892. doi: 10.1371/journal.pone.0071892.

11. Moura AC, Lazzari VM, Agnes G, Almeida S, Giovenardi M, Veiga AB. Transcriptional expression study in the central nervous system of rats: what gene should be used as internal control? Einstein (São Paulo). 2014;12(3):336-41. doi: 10.1590/S1679-45082014AO3042.

12. Swijsen A, Nelissen K, Janssen D, Rigo JM, Hoogland G. Validation of reference genes for quantitative real-time PCR studies in the dentate gyrus after experimental febrile seizures. BMC Res Notes. 2012;5. doi: 10.1186/1756-0500-5-685.

13. Tanic N, Perovic M, Mladenovic A, Ruzdijic S, Kanazir S. Effects of aging, dietary restriction and glucocorticoid treatment on housekeeping gene expression in rat cortex and hippocampus-evaluation by real time RT-PCR. J Mol Neurosci. 2007;32(1):38-46. doi: 10.1007/s12031-007-0006-7.
