# Supplementary material for: With Reference to Reference Genes: A Systematic Review of Endogenous Controls in Gene Expression Studies
Source: PLoS One. 2015 Nov 10;10(11):e0141853. doi: 10.1371/journal.pone.0141853 (PMC4640531; doi:10.1371/journal.pone.0141853)
Supplement: S5 Table — Differences in the number of RGs used in each of the seven categories of study species (rodent = rodent model species and livestock = livestock mammal). (DOCX) [file pone.0141853.s006.docx]

**Table S2**.

| **Dunn's multiple comparisons test** | **Mean rank difference** | **Significant?** |
| --- | --- | --- |
| Human vs Rodent | 41.33 | No |
| Human vs Livestock | 36.46 | No |
| Human vs Fish | 91.97 | Yes |
| Human vs Other mammal | 5.144 | No |
| Human vs Bird | 98.92 | No |
| Human vs Primate | 18.56 | No |
| Rodent vs Livestock | -4.872 | No |
| Rodent vs Fish | 50.64 | No |
| Rodent vs Other mammal | -36.18 | No |
| Rodent vs Bird | 57.59 | No |
| Rodent vs Primate | -22.77 | No |
| Livestock vs Fish | 55.51 | No |
| Livestock vs Other mammal | -31.31 | No |
| Livestock vs Bird | 62.46 | No |
| Livestock vs Primate | -17.9 | No |
| Fish vs Other mammal | -86.82 | No |
| Fish vs Bird | 6.951 | No |
| Fish vs Primate | -73.41 | No |
| Other mammal vs Bird | 93.77 | No |
| Other mammal vs Primate | 13.42 | No |
| Bird vs Primate | -80.36 | No |
